# Validity of clinically significant change classifications yielded by Jacobson-Truax and Hageman-Arrindell methods

**DOI:** 10.1186/s12888-016-0895-5

**Published:** 2016-06-06

**Authors:** Fiona R. Ronk, Geoffrey R. Hooke, Andrew C. Page

**Affiliations:** School of Psychology, The University of Western Australia, 35 Stirling Hwy, Crawley, 6009 Australia; Perth Clinic, Perth, Western Australia

**Keywords:** Clinical significance, Validity, Outcomes, Recovery, Quality of life

## Abstract

**Background:**

Reporting of the clinical significance of observed changes is recommended when publishing mental health treatment outcome studies and is increasingly used in routine outcomes monitoring systems. Since recovery rates vary with the method chosen, we investigated the validity of classifications of clinically significant change when the Jacobson-Truax method and the Hageman-Arrindell method were used.

**Methods:**

Of 718 inpatients who completed the Depression Anxiety Stress Scales (DASS-21) and Quality of Life Enjoyment and Satisfaction Questionnaire at admission and discharge to a psychiatric clinic, 355 were invited (and 119 agreed) to complete the questionnaires and the Recovery Assessment Scale six weeks post discharge.

**Results:**

Both the JT and HA methods showed comparably good validity when referenced against the other indices. Clinically significant change on the DASS-21 was related to a greater consumer-based sense of recovery, greater perceived quality of life, and fewer readmissions to hospital within 28 days of discharge.

**Conclusions:**

Since there was found to be no advantage to using one method over another when recovery is of interest, the simpler JT method is recommended for routine usage.

## Background

Clinical significance categorisations aim to provide a meaningful classification of treatment outcomes [1, 2]. The most widely used calculation for clinical significance is the Jacobson-Truax method [3, 4] which considers the reliability of the change made (Reliable Change Index; RCI) in the context of the overall distribution that the patient is likely to belong to post-treatment (functional or dysfunctional). Clients moving reliably into the functional distribution are *recovered*. Clients have *improved* if they have made a reliable change but remain in the dysfunctional population, *unchanged* if they have not made a reliable change, and *deteriorated* if they have reliably worsened.

Given recommendations to report rates of clinically significant change, it is important that classifications are valid [5]. Supporting the ecological validity of the Jacobson-Truax (JT) method, clinically significant change on the Symptom Check List-90 Revised (SCL-90R) [6] relates to client’s satisfaction with therapy [7] and client and therapist-rated change [8]. The convergent validity of classifications of clinically significant change for depressed patients across different depression measures has also been demonstrated [9]. Likewise, clients who made a clinically significant change on the Outcomes Questionnaire-45 (OQ-45) [10] also made clinically significant change on the SCL-90R, the Social Adjustment Rating Scale, the Inventory of Interpersonal Problems, and the Quality of Life Inventory [11]. Newnham, Harwood, and Page [12] determined that clinically significant change on the Medical Outcomes Short Form Questionnaire (SF-36) [13] was associated with a greater perceived quality of life, as well as greater clinician-rated functioning. Furthermore, Wise [14] demonstrated that 56 % of substance abuse clients who made a reliable change on the SCL-90R had a clinically meaningful change in the percentage of days abstinent from substances. Ronk and colleagues [15] demonstrated that when clinical significance based on the JT method is assessed using different measures related to depression (Quality of Life Enjoyment Scale; Depression scale of the DASS-21; and SF-36 Mental Health Scale), the results are largely convergent. Therefore, these findings support the ecological validity of clinical significance classifications for the JT method of calculating the clinical significance.

### Potential alternatives to JT method

However, there exist several other methods to classify the clinical significance of a treatment outcome, including the Gulliksen Lord Novick method (GLN) [16, 17], the Nunnally-Kotsch method (NK) [18], the Edwards-Nunnally method (EN) [19], the Hageman-Arrindell method (HA) [20] and Hierarchical Linear Modelling (HLM) [21]. McGlinchey, Atkins, and Jacobson [22] found that the HA method classified clients significantly differently to the JT, GLN, and EN methods. The HA method was less sensitive since a greater amount of client change was required for a client to be considered reliably improved. Similarly, Ronk, Hooke, and Page [15] found that while the JT, GLN, NK, EN calculation methods yielded similar rates of clinically significant change, the HA method produced consistently distinct classifications. Therefore, the HA method is more conservative in assigning classifications of *recovered* to patients than other methods.

The reason for differences between the classification rates lies in the method of calculations. The JT method classifies a client’s outcome based on the reliability of the pre- to post-treatment change and whether or not the client has moved from the dysfunctional population to the functional population. The calculation uses clients’ observed scores, which contain measurement error. The HA method attempts to correct regression to the mean by using an approximation of true scores rather than observed scores. In addition, while the JT method uses a cut-off score to separate the functional and dysfunctional distributions, the HA method uses a cut-off index score which allows users to determine that a client has passed the cut-off score in the correct direction with 95 % confidence.

These differences between rates of recovery based on the JT and HA methods of classifying change need to be explored. However, as previously stated by Hsu [23], one method cannot be recommended over another based purely on higher or lower rates of classifications of clinically significant change. It is important to determine whether one method’s recovered clients experience changes in other areas of importance, such as quality of life, that reasonably correspond with the concept of recovery, when compared to other classification methods (see [22]). Thus, analyses will only be conducted using the JT and HA methods, since the remaining three methods explored in McGlinchey et al., [22] and Ronk et al. [15] were largely similar to the JT method.

### Recovery evaluation

While in clinical research symptom reduction is often synonymous with recovery, ‘consumer-based recovery’ captures the notion that there are many facets to recovery, including hope, healing, empowerment, self-identity, pursuing meaningful goals, developing connections with others, and having a sense of control [24, 25]. This definition of recovery posits that a focus on symptom reduction alone is too narrow, as clients who report severe symptoms can still experience improvements in other aspects of their lives [26, 27] and vice versa. Therefore, in the present study the first recovery evaluation variable is a consumer-based measure of recovery; the Recovery Assessment Scale (RAS) [28].

Another domain associated with the consumer-based conceptualisation of recovery is quality of life [29]. Although symptoms and quality of life are not mirror images on one another, an increase in symptoms relates to a decrease in quality of life for patients with Major Depressive Disorder [30]. If the inverse of this is also true, and clinically significant change on a symptom measure relates to significant improvements in perceived quality of life, then this will provide convergent validity for classifications of the *recovered* category. Therefore, the second recovery evaluation measure chosen is the Quality of Life Enjoyment and Satisfaction Questionnaire (Q-LES-Q) [31, 32].

In addition, it is important to include an objective indicator of treatment outcome. Whether or not a patient has been readmitted to an inpatient facility soon after their discharge has a logical relationship to outcome. Readmission to hospital, specifically within the 28 day period following discharge, is used as a national clinical indicator of the quality of care [33]. Readmission to hospital within 28 days is considered a poor outcome associated with more severe symptoms [34, 35], and prior hospital admissions [36]. Specifically, being readmitted to hospital within 28 days is not consistent with that person being considered as recovered [37, 38].

### Current study

The focus of the current study is on the categorisation of patients as *recovered*, which is defined according to statistical methods for reporting clinical significance as both (a) making a statistically reliable change during treatment; and (b) belonging to the ‘functional’ population at post-treatment. Firstly, we aim to examine the validity of the JT method for assessing clinically significant change by exploring the relationship between classifications of *recovered* and three variables related to the concept of recovery. It is necessary to explore the links between clinical significance classifications and these criterion measures before any further assumptions can be made about the validity of the clinical significance methodology. It is hypothesised that those patients who are classified as *recovered* by either the JT or HA methods, when compared to those who are not classified as *recovered*, will:

- score higher on the Recovery Assessment Scale (RAS) [39, 40] indicative of a greater sense of consumer-based recovery;
- score higher on the Quality of Life Enjoyment and Satisfaction Questionnaire (Q-LES-Q) [31], indicative of a greater perceived enjoyment and satisfaction with life; and
- have lower rates of readmission to hospital within 28 days of their discharge, indicative of a more successful post-discharge period.

Secondly, if there is an association between a clinically significant change and our three criterion measures, we aim to determine whether one method demonstrates more convergent validity than another.

## Method

### Participants and procedure

Participants were 718 consecutively admitted patients with complete data discharged from a private psychiatric hospital between April 2011 and January 2012. The mean age was 42.9 years (*SD* = 15.1) and the mean length of hospital stay was 17.4 days (*SD* = 14.8). Married patients made up 50.3 % of the sample, 33.2 % were single, and 16.5 % were separated, divorced, or widowed. Participants were given diagnoses by their treating psychiatrist. The sample consisted of patients with primary diagnoses of mood (56.1 %), anxiety (19.4 %), substance use (13.7 %), and psychotic disorders (5.9 %) as well as other diagnoses (4.9 %).

While in hospital, patients complete a range of group therapies led by clinical psychologists and occupational therapists including cognitive behavioural therapy, interpersonal therapy, and structured activity-based therapy while under the care of nursing staff and their psychiatrist. As part of routine quality assurance at the hospital, patients were invited to complete questionnaire measures at both admission and discharge. Participants provided informed consent and the study had ethical approval (#2557).

Patients completed the DASS-21 and Q-LES-Q at admission and discharge. A total of 718 patients discharged during the period from April 2011 and January 2012 completed both measures at admission and at discharge. These data were used to assess clinical significance of change from pre- to post-treatment as measured by the DASS-21 as well as quality of life score at discharge and readmission to hospital within 28 days of discharge. A cohort of 355 patients discharged during the first half of the study period was invited to complete the DASS-21 and RAS six weeks after discharge. The total response rate was 41.1 %, which compares favourably with other mail-out surveys. Only cases with complete data (*n* = 119) were used in the analyses of scores six weeks post-discharge. Age differed significantly between responders (*M* = 48.0 years; *SD* = 15.7) and non-responders (*M* = 40.4 years; *SD* = 15.0); *t*(353) = 4.68, *p* < .05. Length of stay in hospital was longer (*M* = 19.2 days; *SD* = 15.8) for patients who responded compared to patients who did not respond (*M* = 16.1 days; *SD* = 12.2); *t*(353) = 2.16, *p* < .05. There were no significant differences between responders and non-responders in symptom severity at admission or discharge and prior admissions to hospital.

### Measures

#### Recovery Assessment Scale (RAS)

Scores on the RAS have high internal consistency (α = .93) and test-retest reliability (α = .88) [28, 41]. The validity of RAS score interpretations demonstrates convergent validity [28, 42] with correlations with other recovery-oriented scales ranging from *r* = .20 - .68. The RAS demonstrates divergent validity from symptom or function-based measures such as the Health of the Nation Outcome Scales (HoNOS) [43, 44]. The RAS originally had 41 items, however Hancock et al. [40] removed 10 items due to poor fit statistics or item redundancy, resulting in a 31-item scale that map more closely to processes associated with consumer based recovery in the literature (e.g., symptom management, a sense of control). Based on results of the Rasch analysis [40], the 31-item version of the RAS with a five-point rating scale (1 = strongly disagree; 5 = strongly agree) was chosen to determine recovery scores in the current study. Scores are summed to form one score representing ‘recovery’, with a minimum possible score of 31 and a maximum possible score of 155. A higher score indicates a stronger experience of recovery.

#### Depression Anxiety Stress Scales 21 (DASS-21)

Lovibond and Lovibond [45] the DASS-21 measures levels of depression, anxiety, and stress. Respondents rate 21 items such as “I felt down-hearted and blue” and “I felt that life was meaningless” on a scale ranging from zero to three. Within each scale, the total score is doubled so that the minimum score is zero and the maximum score is 42. The scores on each scale have high internal consistency (α = .88 for Depression; α = .82 for Anxiety and α = .90 for Stress; [46]) and the interpretations of the construct demonstrate good convergent and discriminant validity [45–48].

#### Quality of Life Enjoyment and Satisfaction Questionnaire- Short Form (Q-LES-Q)

Endicott et al. [31] the Q-LES-Q is a 14 item self-report scale assessing quality of life across domains such as physical health, and household activities. Respondents rate their satisfaction with each domain on a 5-point scale. Item scores are added and transformed onto a scale ranging from 0 to 100, with higher scores indicative of higher perceived quality of life. Scores on the Q-LES-Q demonstrate high internal consistency (>.90) and test-retest reliability (.63–.89), and the interpretations of the scores show good construct validity [30, 32, 49].

### Clinical significance calculation methods

The current study used the Jacobson-Truax method of clinical significance classification [3, 4] and the Hageman Arrindell method of clinical significance classification [19]. When using the JT the cut-off between the dysfunctional population and the functional population can be calculated using one of three formulas. The present study used cut-off ‘C’ to represent the cut-off between the functional and dysfunctional population as recommended by Hsu [50]. In addition to classifications of clinical significance made using scores from the time of pre-treatment to post-treatment, classifications were also calculated using pre-treatment scores and scores at six weeks post-treatment.

### Data analysis

Independent sample *t*-tests and corresponding measure of effect size, Cohen’s *d*, will be used to evaluate the difference in RAS scores and quality of life scores for those patients who make a clinically significant change on the DASS-21 and those who do not. Chi-squared analysis (*χ*^*2*^) and corresponding measure of effect size, phi (ɸ) will be used to assess the difference in readmission rates within 28 days between those patients who make a clinically significant change on the DASS-21 and those who do not. Following Cohen [51], the conventions for small, medium and large effect sizes are respectively .20, .50 and .80 for Cohen’s d, and .10, .30 and .50 for Φ (and the Pearson correlation coefficient, r).

## Results

Scores for each scale of the DASS-21 decreased between pre-treatment and post-treatment, and quality of life increased (Table 1). DASS-21 scales at pre-treatment, post-treatment, and between change scores were moderately inter-correlated. Pre-treatment correlations were significant (*p < .01)*; *r*_(Dep_ & _Anx)_ = .52, *r*_(Dep_ & _Str)_ = .57, and *r*_(Anx_ & _Str)_ = .69. Post-treatment correlations were significant (*p < .01)*; *r*_(Dep_ & _Anx)_ = .70, *r*_(Dep_ & _Str)_ = .78, and *r*_(Anx_ & _Str)_ = .74. Correlations between change scores were also significant (*p < .01)*; *r*_(Dep_ & _Anx)_ = .59, *r*_(Dep_ & _Str)_ = .68, and *r*_(Anx_ & _Str)_ = .69.

**Table 1:** Means and standard deviations (in parentheses) for the DASS-21, Q-LES-Q and RAS at pre-treatment, post-treatment, and six weeks post-treatment

		DASS-21 scale			Q-LES-Q	RAS
	*n*	Depression	Anxiety	Stress		
Pre-treatment	718	28.27 (11.84)	20.53 (10.85)	26.96 (10.24)	32.59 (17.42)	-
Post-treatment	718	12.82 (10.79)	10.18 (8.95)	13.63 (9.69)	52.97 (18.69)	-
Six weeks post-treatment	119	12.66 (11.69)	9.78 (9.16)	14.13 (10.53)	-	119.15 (18.26)
Range of measure		0 – 42^a^	0 – 42^a^	0 – 42^a^	0 – 100^b^	31 – 155^b^

*DASS-21* depression anxiety stress scales – 21, *Q-LES-Q* quality of life enjoyment and satisfaction questionnaire, *RAS* recovery assessment scale

^a^higher scores reflect more negative functioning. ^b^higher scores reflect more positive functioning

When patients are classified using the JT method, there are higher rates of clinically significant change than when the HA method is used (Table 2). This suggests that the HA method is more stringent in its classification of recovery. Classifications of deterioration yield identical proportions with both methods. Of the 718 patients discharged, 64 (8.9 %) were readmitted within 28 days of their discharge from hospital.

**Table 2:** Percentage of patients classified into each clinical significance category by the Jacobson-Truax (JT) method and the Hageman-Arrindell (HA) method based on DASS-21 scale scores calculated across two time periods

	DASS-21 Depression scale		DASS-21 Anxiety scale		DASS-21 Stress scale	
	JT method	HA method	JT method	HA method	JT method	HA method
Pre-treatment to post-treatment (*n* = 718)						
Recovered	57.5 %	42.1 %	36.8 %	19.1 %	58.9 %	46.7 %
Improved	14.9 %	35.9 %	14.6 %	48.1 %	11.6 %	29.9 %
Unchanged	25.5 %	19.9 %	46.4 %	31.3 %	27.1 %	21.0 %
Deteriorated	2.1 %	2.1 %	2.2 %	1.5 %	2.4 %	2.4 %
Pre-treatment to six weeks post-treatment (*n* = 119)						
Recovered	49.5 %	37.8 %	36.1 %	18.5 %	56.3 %	47.9 %
Improved	11.8 %	29.4 %	14.3 %	47.0 %	10.1 %	23.5 %
Unchanged	34.5 %	28.6 %	47.9 %	32.8 %	23.5 %	18.5 %
Deteriorated	4.2 %	4.2 %	1.7 %	1.7 %	10.1 %	10.1 %

### Recovery assessment scale

Of 119 patients who completed the RAS six weeks following discharge from hospital (Table 3), between 18.5 and 56.3 % of patients were classified as recovered, depending on which DASS-21 scale a patient was measured on and with which clinical significance calculation method. Patients who were classified as recovered on each scale of the DASS-21 according to both the JT and HA methods scored significantly higher on the RAS than those who made no clinically significant change. A similar pattern was found when patients who were classified as recovered according to the HA method were compared with those who were not. This suggests that both the JT and HA methods for evaluating clinically significant change (i.e., a classification of recovered) demonstrate construct validity, as clinically significant change on a symptom measure, the DASS-21, is related to higher scores on the RAS, representative of a more positive perception of ‘consumer-based’ recovery.

**Table 3:** Descriptive statistics for patient scores on the recovery assessment scale who have been classified as recovered or not recovered based on each DASS-21 scale using the JT and HA calculation methods

	Classifications based on JT method			Classifications based on HA method		
	Depression	Anxiety	Stress	Depression	Anxiety	Stress
Classified as recovered						
*n*	59	43	67	45	22	57
Mean	126.79	124.65	125.42	130.10	131.77	126.12
SD	14.24	17.52	13.97	13.90	17.34	14.59
Not classified as recovered						
*n*	60	76	52	74	97	62
Mean	111.65	116.04	111.08	112.50	116.29	112.75
SD	18.76	18.04	20.02	17.42	17.29	19.04

### Quality of life

Of 718 patients who completed the Q-LES-Q at post-treatment (Table 4), between 19.1 and 58.9 % were classified as having achieved a clinically significant change, depending on which DASS-21 scale they were assessed on and clinical significance classification method. Perceived quality of life was greater for patients classified as recovered by the JT and HA methods than those who were not. These findings support the construct validity of clinically significant change as calculated by both the methods.

**Table 4:** Descriptive statistics for patient scores on the quality of life enjoyment and satisfaction scale who have been classified as “Recovered” or “Not Recovered” based on each DASS-21 scale using the JT and HA calculation methods

	Classifications based on JT method			Classifications based on HA method		
	Depression	Anxiety	Stress	Depression	Anxiety	Stress
Classified as “recovered”						
*n*	413	264	423	302	137	335
Mean	58.84	60.79	58.65	63.03	66.28	61.28
SD	15.73	16.94	16.23	14.87	16.11	15.53
Not classified as “recovered”						
*n*	305	454	295	416	581	383
Mean	45.03	48.43	44.84	45.68	49.84	45.71
SD	19.46	18.16	19.01	17.78	17.87	18.20

### Readmission to hospital within 28 days of discharge

**Fig. 1:**
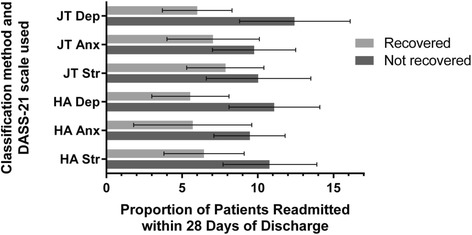
Proportion of patients readmitted within 28 days of discharge who are considered recovered and not recovered by the JT and HA methods used with the DASS-21 scale scores. Error bars represent 95 % confidence intervals

A significantly higher proportion of patients who were not considered recovered at discharge were readmitted within 28 days than those who were considered recovered by the JT method with the Depression scale (*χ*^*2*^(1) = 9.80, *p* = .002, ɸ = .117; Fig. 1), the HA method with the Depression scale (*χ*^*2*^(1) = 6.93, *p* = .008, ɸ = .098), and the HA method with the Stress scale (*χ*^*2*^(1) = 4.259, *p* = .039, ɸ = .077). The remaining classification methods yielded no significant differences between readmission rates for recovered compared to non-recovered patients. Since patients who were not considered to have made a clinically significant change on the Depression scale were approximately twice as likely to be readmitted within 28 days of discharge than those patients whose change had been considered clinically significant, this provides support for the construct validity of recovery as evaluated by both calculation methods but only when classifications are based on certain DASS-21 scores.

## Discussion

When patients who received a classification of *recovered* at post-treatment (calculated using the JT method with DASS-21 scores) were compared to those who were not considered *recovered*, the *recovered* patients had significantly higher RAS scores, indicative of a more positive consumer-based sense of recovery, and significantly higher Q-LES-Q scores, indicative of a greater perception of life enjoyment and satisfaction. The rate of hospital readmission within 28 days of discharge was significantly lower for those considered *recovered* according to the JT method with the Depression scale, and the HA method with the Depression and Stress scales. These findings provide further support that classifying patients as *recovered* according to the Jacobson-Truax method of clinical significance calculation has construct validity when used with a symptom measure.

Despite the differences in recovery rates between the more lenient, popular JT method and the more conservative, less commonly used HA method [15, 22], a comparison of effect sizes did not uncover any significant differences between the methods. This suggests there are no meaningful differences between how the methods capture the construct of recovery as conceptualised by the variables chosen in the current study. Therefore, we echo the recommendation [1] that the JT method continue to be used since it is the most commonly used and simplest to calculate.

### Limitations

It could be argued that since the current study was correlational in nature, it was not possible to determine which method was better ‘calibrated’ towards recovery. This is true, however the issue of calibration is an arbitrary one, since the category of recovered has demonstrated meaning from the perspective of both the patient and treatment provider. Whether the ‘true’ rate of recovered patients is indeed higher or lower than that determined by the JT method is not relevant if the arbitrary categories have meaning.

Although we can conclude here the JT and HA methods appear to have similar conceptualisations of the category of *recovered*, the current study does not allow for any comment about the validity of the categories of *improved, unchanged,* or *deteriorated.* Further research is required to determine the relationship between belonging in each of these categories and scores on relevant behavioural or functional indices, as well as individual client factors. For example, it may be that clients who are *unchanged* during treatment have lower scores on readiness to change measures. If this is the case, then clinicians could employ specific techniques such as motivational interviewing [52] for those clients who score low on a readiness to change measure at pre-treatment, to increase their chances of making a reliable or clinically significant change during treatment.

Of particular concern to clinicians are those people who deteriorate during treatment. Validity studies need to focus on these clients, as they are not often included in assessments of clinical significance. One reason for their lack of inclusion in such research may be the typically low proportion of clients who receive this classification. Of course, having very few deteriorators in a sample is desirable from a clinical perspective, but makes it more difficult to explore the correlates of deterioration, as in the current study. Since the present sample consisted of inpatients that generally score high on symptom measures, the chances of increasing symptoms enough to achieve a reliable deterioration are lower than in outpatient samples. An added complexity in regards to deteriorators is that they are not a homogenous group; the negative, reliable change required to be classified as *deteriorated* can occur anywhere along the range of the outcome measure. For example, a deterioration based on movement from the normal range to the mild range is qualitatively different to a deterioration based on movement from the severe range to the extremely severe range of a symptom measure. It therefore follows that correlates of deterioration may be equally as heterogeneous. Larger samples of patients are required to meaningfully explore the correlates of this form of patient change. Methods employed in the feedback literature [53–56] could then be used to predict which patients are “at-risk” of deteriorating, allowing clinicians to intervene during treatment. In addition to these concerns, it is relevant to note that it is not always possible or practical to calculate clinical significance. That is, some scales do not (and sometimes cannot) have relevant normative information and reliability estimates and for low prevalence mental health conditions the case for applicability needs to be made. Likewise, while the present paper has explored to some degree what is perceived as ‘clinically significant change,’ it is possible that the classification may vary depending on the perspective of the rater (i.e., client, clinician, carer, service provider, etc).

The use of readmission to hospital within 28 days of discharge as an index of recovery has limitations. A small proportion (5–8 %) of patients who are classified as *recovered* are readmitted to hospital within 28 days, and not all patients who worsen (and perhaps require readmission) will be readmitted. Furthermore, patients who require further treatment do not always require this for the same reasons as a prior admission, nor do they always seek it from the same facility. Despite this, evaluating readmission is an objective, routinely used clinical indicator of the quality of an episode of mental health care that can provide useful information. McGlinchey et al. [22] stated that if clinical significance classifications are valid, then they should mean something in practical terms, regarding whether an individual will remain recovered over time. In the current sample, although the rates of readmission were lower for patients classified as *recovered* than for those who were not, being assigned this classification did not remove the possibility of readmission altogether. Future research should explore the factors associated with hospital readmission subsequent to making a clinically significant change during the initial admission.

Since participants in the current study had diagnoses predominantly of mood and anxiety disorders, the current findings should generalise well to most psychiatric populations. However, for populations with mood and anxiety disorders, scores derived from self-report measures (e.g., Q-LES-Q) may be influenced by patients’ current mood, their level of insight, or recent life events [57]. This issue is present in all self-report studies in psychiatric samples, and relates also to the symptom measures on which clinically significant change is measured. Furthermore, the treatment provided to patients in the current study was voluntary within an inpatient setting, therefore further research may be required to explore whether the validity of clinical significance classifications is supported in those populations where treatment is involuntary, or provided in outpatient settings. Finally, the patients who responded in the current study were older and had longer lengths of stay than those who did not respond; several hypotheses could explain this difference. However, since a focus of the study was upon the comparison of two methods of calculating clinical significance, the differences between respondents and non-respondents were not considered relevant; the more important issue was that the same patients were included in each comparison analysis.

## Conclusions

Classifying change into valid clinical significance categories following mental health treatment allows treatment providers to evaluate treatment effectiveness, provide valid feedback, and allows for ongoing quality improvement. Current findings suggest that classifications of clinically significant change made using the DASS-21 demonstrate ecological, construct validity, since classifications of *recovered* align with more positive perceptions of consumer-based recovery, greater perceived life enjoyment and satisfaction, and a lower chance of being readmitted to hospital with 28 days of discharge. These results together with validity findings in the extant literature suggest that the commonly used Jacobson-Truax method of classifying clinically significant change does exhibit validity, and therefore the recommendation that clinical significance classifications are reported in every outcome study is warranted. Additionally, there was no discernible advantage to using the HA method over the JT method, therefore the use of the simpler, JT method, is recommended. The JT methodology provides an easy, fast, and most importantly, ecologically valid way to approximate the meaningfulness of a clients’ change.

## Abbreviations

(Q-LES-Q), quality of life enjoyment and satisfaction questionnaire; DASS-21, depression anxiety stress scales; EN, Edwards-Nunnally method; GLN, Gulliksen Lord Novick method; HA, Hageman-Arrindell method; HLM, hierarchical linear modelling; HoNOS, health of the nation outcome scales; JT, Jacobson-Truax method; NK, Nunnally-Kotsch method; OQ-45, outcomes questionnaire-45; RAS, recovery assessment scale; RCI, reliable change index; SCL-90R, symptom check list-90 revised; SF-36, medical outcomes short form questionnaire
